# Antimicrobial Photodynamic Therapy Protocols on *Streptococcus mutans* with Different Combinations of Wavelengths and Photosensitizing Dyes †

**DOI:** 10.3390/bioengineering6020042

**Published:** 2019-05-10

**Authors:** Elisabetta Merigo, Stefania Conti, Tecla Ciociola, Maddalena Manfredi, Paolo Vescovi, Carlo Fornaini

**Affiliations:** 1 Laboratoire MICORALIS (MICrobiologie ORALe, Immunothérapie et Santé) EA7354, Université Nice Sophia Antipolis, UFR Odontologie, Avenue des Diables Bleus, 06000 Nice, France; elisabetta.merigo@unice.fr; 2 Department of Medicine and Surgery, University of Parma, Via Gramsci 14, 43126 Parma, Italy; stefania.conti@unipr.it (S.C.); tecla.ciociola@unipr.it (T.C.); maddalena.manfredi@unipr.it (M.M.); paolo.vescovi@unipr.it (P.V.); 3 GAEM, Group of Applied ElectroMagnetics, Department of Engineering and Architecture, University of Parma, Viale G. P. Usberti 181/A, 43124 Parma, Italy

**Keywords:** photodynamic therapy, *Streptococcus mutans*, laser, wavelengths, photosensitizers

## Abstract

The aim of the study is to test the application of different laser wavelengths, with and without different photosensitizing dyes on different types of cultures. Laser irradiation was realized on *Streptococcus mutans* in both solid and liquid culture media in continuous mode at three different fluences (10, 20, and 30 J/cm^2^) with a red diode (650 nm) with toluidine blue dye, a blue-violet diode (405 nm) with curcumin dye, and a green diode (532 nm) with erythrosine dye. Without a photosensitizer, no growth inhibition was obtained with the red diode at any fluence value. Inhibition rates of 40.7% and 40.2% were obtained with the blue diode and green diode. The blue diode laser used with curcumin obtained results in terms of growth inhibition up to 99.26% at a fluence of 30 J/cm^2^. The red diode laser used with toluidine blue obtained results in terms of growth inhibition up to 100% at fluences of 20 and 30 J/cm^2^. The KTP (potassium-titanyl-phosphate) laser used with erythrosine was able to determine a complete growth inhibition (100%) at the different fluence values. The combination of a laser and its proper color may dramatically change the results in terms of bactericidal effect. It will be interesting to confirm these data by further in vivo studies.

## 1. Introduction

Recently, we observed an accelerated increase in antibiotic-resistant infections. Despite therapy, infectious diseases remain the main cause of mortality, with bacterial infections causing 17 million deaths globally [1]. 

The United Kingdom (UK) Department of Health recently published a report describing antibiotic resistance as the cause of 700,000 deaths per year, estimating that this figure will increase up to 10 million per year in 2050 [2]. This is the main reason why it is critical to focus international research on the search for new antibacterial drugs which are able to prevent and eradicate bacterial resistance. 

Over 100 years ago, Oskar Raab discovered the basis of photodynamic therapy (PDT), a therapeutic technique using cytotoxic action for the treatment of infectious diseases and tumors that has been in use since the 1970s [2,3]. In PDT, the cytotoxic effect is achieved through the local application or systemic administration (oral or intravenous) of photosensitizing agents followed by irradiation of visible light with an emission spectrum appropriate for the absorption spectrum of the photosensitizer in the presence of oxygen [4]. 

In order to make a photodynamic reaction occur, three basic elements are required [5]:

1. A photosensitizing agent that is a photosensitive molecule able to localize itself in a cell or in a target tissue;
2. A light source with specific wavelengths required to activate the photosensitizing agent;
3. Molecular oxygen, essential for the formation of reactive oxygen species (ROS).

Many organic molecules of biological origin can act as photosensitizers as they present a good quantum yield of triplet formation and an excited state with a relatively long lifetime (up to hundreds of microseconds). In fact, absorbed light, only when coupled with a proper wavelength and in the presence of oxygen, can define the transition of the photosensitizer to its photoactive triple state, producing cytotoxic radicals (type 1 reaction) or cytotoxic singlet oxygen and free radicals (type 2 reaction). The cytotoxic molecules produced can ultimately induce cell death.

The requirements of an optimal photosensitizer are multiple; it should be non-toxic and show local toxicity only after activation with light and it should have highly selective accumulation and a high quantum yield of singlet oxygen production [6,7].

Among the first molecules used as photosensitizing agents of living organisms with visible light, there were some agents of natural origin, such as porphyrins [5].

Porphyrins, similar to all chromophores, undergo electronic excitation following the absorption of a quantum of light, passing by the fundamental electronic state to a higher level—the excited singlet [8].

The absorption spectrum of these molecules varies from 400 to 700 nm. For this reason, porphyrins are optimal photosensitizers for applications in this field [9].

PDT requires a light source that activates the photosensitizer by exposure to low-power, visible light at a specific wavelength. Most photosensitizers are activated by red light between 630 and 700 nm [10].

Currently, it is more common to use diode lasers, which are low-cost and easy to handle and transport, as well as LED (Light Emitting Diodes) lights [11,12,13,14,15].

In medicine and dentistry, PDT for antimicrobic purposes is used for different types of applications with different types of photosensitizers and lasers [16]. It has also been used recently with systems based on LED technology [16,17,18]. In dentistry, for example, there is growing interest in the possible application of laser PDT in the treatment of periodontitis and peri-implantitis and in endodontics [19,20,21,22]. In antitumor treatments using PDT, the photosensitizer is mainly applied by systemic administration, whereas in the case of oral antimicrobial PDT, topical application to the target zone is the most widely used route of in vivo administration, for example, at the intrasulcular site for periodontal purposes or into root canals for endodontic applications.

The aim of this study was to test, in various photodynamic therapy protocols, the application of different laser wavelengths in combination, or not, with different photosensitizing dyes in different types of cultures (liquid or solid) of *Streptococcus mutans*. *Streptococcus mutans* is a Gram-positive aerobic and facultative anaerobic bacterium belonging to α-hemolyzing streptococci. It was first described by Clarke in 1924, and thereafter was classified as *Streptococcus viridans* [23]. Physiologically, it occurs in the human mouth, entering as a natural bacterial flora. *Streptococcus mutans*, along with *L. acidophilus* and other bacteria, constitute one of the main etiological factors in the formation of dental caries. In the case of gingivitis, this bacterium can pass through damaged mucous membranes into the bloodstream, causing transient bacteremia and infective endocarditis.

We chose this bacterium because it is easy to manipulate and it is implicated in different types of oral pathological conditions.

## 2. Materials and Methods 

### 2.1. Microbial Strain and Culture Conditions 

The reference strain *S. mutans* was grown on Mueller-Hinton agar plates at 37 °C for 24 h. Cells were collected via centrifugation and washed three times with PBS (Phosphate-buffered saline). Cells were counted using a Burker hemocytometer (Emergo, Landsmeer, the Netherlands), and the cell suspension was properly diluted in saline to achieve a final concentration of 5 × 10^7^ cells/mL.

### 2.2. Dyes and Laser Sources

Each of the experimental conditions and protocols was realized in duplicate for agar plates and triplicate for Eppendorf tubes on cultures of *Streptococcus mutans*. We realized every laser application of *Streptococcus mutans* cultured on Mueller-Hinton agar plates or suspended in saline solution in Eppendorf tubes.

Erythrosine and toluidine blue were dissolved in distilled sterile water and curcumin in dimethyl sulfoxide (DMSO) at 20 mM concentration. The final working concentration was 100 μM for erythrosine and curcumin and 10 μM for toluidine blue. We chose the final working concentration on the basis of previous experience and studies [18].

Three laser prototypes, characterized by a Gaussian intensity distribution (TEM 00) in a laser beam, the optical power of which was measured with a power meter (PM-200, Thorlabs), were used for this study. These three prototypes were a red diode laser (spot size 0.2 cm^2^), wavelength 650 nm, coupled with toluidine blue; a blue-violet diode laser (spot size 0.2 cm^2^), wavelength 405 nm, coupled with curcumin; and a green diode laser (spot size 0.785 cm^2^), wavelength 532 nm, coupled with erythrosine.

The choice of these photosensitizers was made on the basis of their optical properties, particularly the absorption coefficient as a function of the laser wavelengths used. In fact, with an absorption maximum of λmax = 635 nm, toluidine blue is ideally suited for irradiation with red lasers [24], while the peak absorption for erythrosine is 532 nm [25]. The best absorption in curcumin is in the region of 420–450 nm [26]. 

Laser irradiation was performed in continuous mode for the different wavelengths. Time irradiation was planned at fluences of 10 J/cm^2^, 20 J/cm^2^, and 30 J/cm^2^ on the basis of the recorded power for each wavelength. The 650 nm diode laser was applied for 307 s, 615 s, and 923 s to plates and Eppendorf tubes with and without toluidine blue. The 405 nm diode laser was applied for 50 s, 100 s, and 150 s to plates and Eppendorf tubes with and without curcumin. The 532 nm diode laser was applied for 95 s, 190 s, and 285 s to plates and Eppendorf tubes with and without erythrosine.

**Figure 1:**
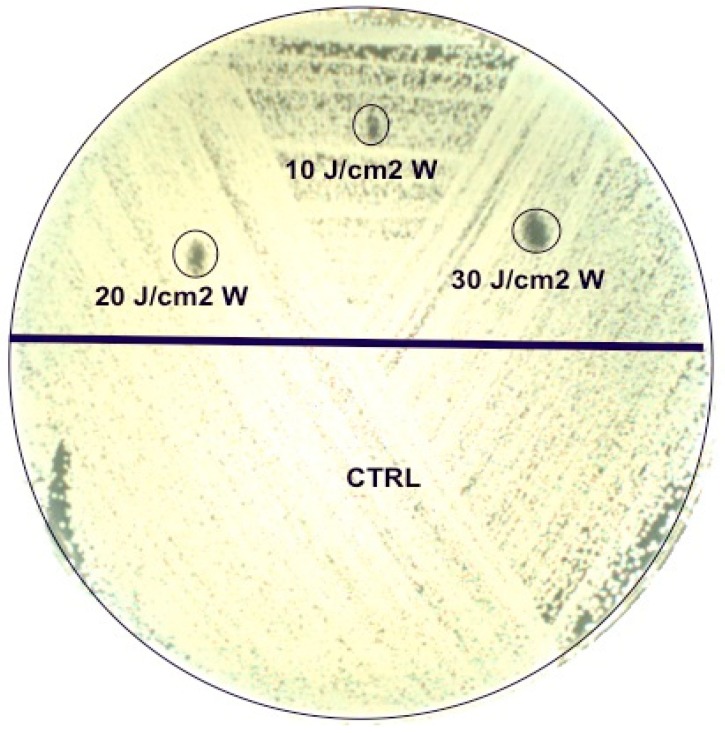
Agar plate irradiation with the three parameters on half of the plate (upper part), using the remaining half as a control.

Agar plates were irradiated with the three parameters on half of the plates, using the remaining half as a control (Figure 1). For suspensions, lasers were applied directly on the top of the tube without the closing cap. For culture plates, the laser handpiece was kept at the same distance by means of a stand and the areas to be exposed to the laser were identified with a paper placed under the plates where the sites of irradiation were drawn. Each assay was carried out in duplicate. 

Agar plates were then incubated at 37 °C in aerobic conditions and observed after one day for the presence of growth inhibition in the irradiated area.

A sample (20 μL) was taken from each Eppendorf tube and streaked onto the entire surface of the agar plate. After one day of incubation at 37 °C in aerobic conditions, colonies were enumerated. The antibacterial effect was evaluated as a percentage reduction of the colony number in comparison to the negative controls (non-irradiated suspensions). 

## 3. Results

The results for the Eppendorf culture are summarized in Table 1, Table 2 and Table 3. 

No inhibition of growth was obtained with the red diode on bacterial suspensions in saline solutions at any fluence value.

The red diode laser used with toluidine blue caused a growth inhibition variable between 99.91% (at a fluence of 10 J/cm^2^) and 100% at fluences of 20 and 30 J/cm^2^.

The zone of inhibition was found only on the plates with toluidine blue where the diameter of the inhibition growth area was 1.6 ± 0.84 mm, 1.9 ± 1.3 mm, and 2.45 ± 2.05 mm at fluences of 10, 20, and 30 J/cm^2^, respectively. In this case, the results were dose-dependent.

The maximum inhibition of growth obtained with the blue diode on bacterial suspensions in saline solutions was 40.7% at a fluence of 20 J/cm^2^.

The same laser parameters applied to bacterial suspensions in saline solution with curcumin caused an inhibition between 78.92% (at a fluence of 10 J/cm^2^) and 99.26% at a fluence of 30 J/cm^2^.

Similar results were obtained in the culture plates. No zone of inhibition was found in plates without curcumin. On the contrary, in plates with curcumin, the diameter of the inhibition growth area was 2.2 ± 0.2 mm, 2.8 ± 0.27 mm, and 3.9 ± 0.14 mm at fluences of 10, 20, and 30 J/cm^2^, respectively, in a dose-dependent manner.

**Figure 2:**
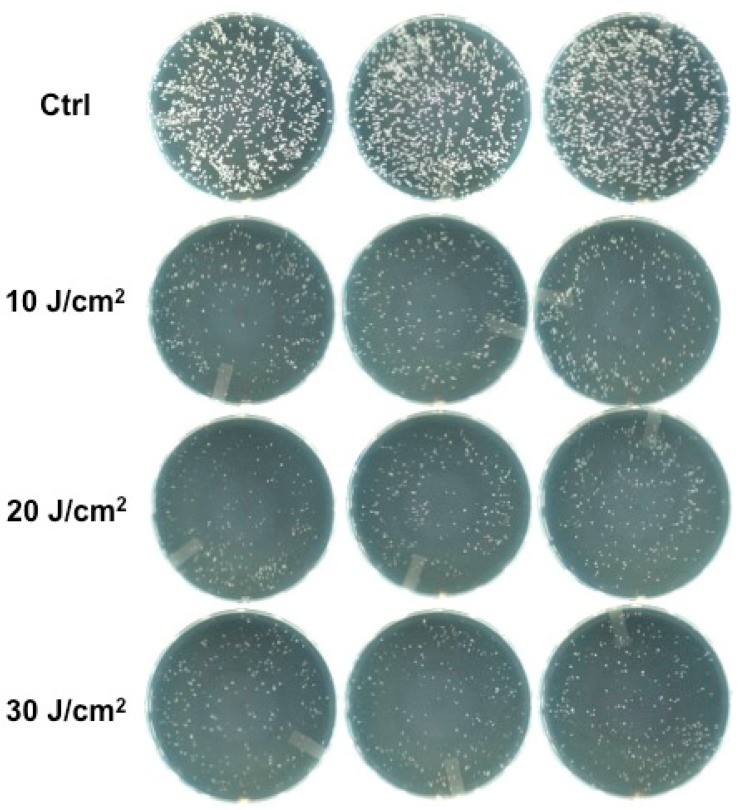
Colonies observed in agar plates after 20 μL from every Eppendorf tube without erythrosine irradiated with the 532 nm green diode laser was taken, streaked on a plate, and incubated for one day.

An inhibition of growth between 32.69% and 40.21% was obtained with the green diode when applied to bacterial suspensions in saline solutions (Figure 2). 

**Figure 3:**
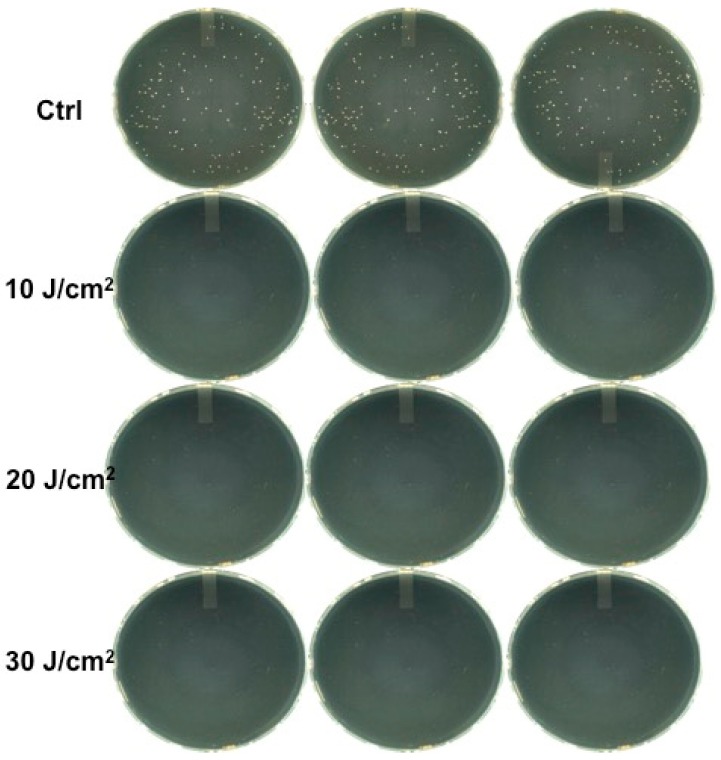
Colonies observed in agar plates after 20 μL from every Eppendorf tube with erythrosine irradiated with the 532 nm green diode laser was taken, streaked on a plate, and incubated for one day.

On the contrary, irradiation with the green diode with erythrosine resulted in complete growth inhibition (100%) at the different fluence values (Figure 3). 

**Figure 4:**
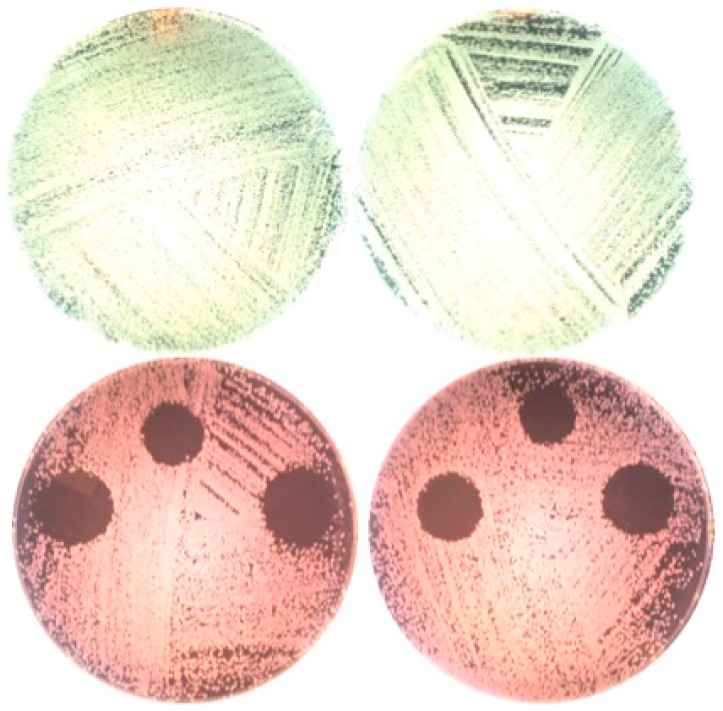
Plates without (upper part) and with (lower part) erythrosine after irradiation with the 532 nm green diode laser and incubation for one day.

The zone of inhibition was only found on plates with erythrosine where the diameter of the inhibition growth area was 13.5 ± 0 mm, 17 ± 1.2 mm, and 17 ± 1.2 mm at fluences of 10, 20, and 30 J/cm^2^, respectively (Figure 4).

## 4. Discussion

This laboratory study evaluated the antimicrobial effect of disinfection protocols performed with three different wavelengths applied with or without photosensitizers in order to understand the possible bactericidal effect of the laser light at the chosen parameters on *S. mutans*, which was chosen as the microbiological marker, because it is a key bacterium in the oral microflora.

The use of a laser with different wavelengths at different parameters on bacterial suspensions in saline solutions did not determine an adequate antibacterial effect, resulting in a level of inhibition of up to 40.69% with the blue diode laser. Moreover, red laser irradiation in bacterial suspensions in saline solutions resulted in a proliferation stimulation of only up to 21% at a fluence of 10 J/cm^2^. 

Conversely, the addition of dyes increased the inhibition values from 78.92% to 99.26% for the blue diode laser, from 99.91% to 100% for the red diode laser, and up to 100% for the green diode laser at any applied fluence. These results are greatly important, particularly for the blue diode laser. In fact, in these experimental conditions, curcumin showed an inhibition of 27% compared with the negative control, and the blue diode laser increased the inhibition of the dye up to 100%. A similar result was obtained for the green diode laser and erythrosine, obtaining 100% inhibition even if, under these experimental conditions, erythrosine alone, without the laser, showed 82% inhibition compared with the negative control. 

Basing the choice of dye concentration on previous works, we chose to use 10 µM toluidine blue and under these experimental conditions the dye showed a very high bacterial inhibition (98.78%). This is a weak point for our study because it is not possible to understand the real effect of the combination of toluidine blue and red laser.

Similar results were found for the culture plates, in which the absence of dye determined the absence of inhibition at all the wavelengths. Conversely, in the culture plates with dyes, the inhibition area was visible in a dose-dependent diameter, with the most effective results demonstrated in plates with erythrosine treated with the green diode laser.

These results are very important, because they highlight, once again, the key role of the choice of the right photosensitizer in PDT protocol for antimicrobial purposes.

Moreover, in this study we chose dyes on the basis of their safety profiles. The products we chose are already available on the market. Toluidine blue is used in medical applications, for example, as vital staining; erythrosine is used for staining in food products; and curcumin is known as a turmeric-derived part of the well-known Indian spice, which is also used for its antioxidant properties [27]. In fact, according to a growing number of studies, curcumin may slow down the development of tumors and it is being studied as a potential source for the prevention of dementia and Alzheimer’s disease, although there are also studies indicating some moderately toxic effects when injected and skin irritation as a side effect [28,29].

The efficacy of curcumin on both Gram-positive and Gram-negative bacteria is proven [30], thanks to its reduction of the adherence properties of *Streptococcus mutans* [27].

The real mechanism for the basis of the antimicrobial action of curcumin combined with visible light is not completely understood at present [31]; however, our results are in agreement with previous results showing the antibacterial properties of curcumin alone, with an increase in antimicrobial action with the addition of blue laser light. The fluences we applied were obtained under blue light for durations between 50 and 150 s and were within the range first described by Dahl and co-workers regarding the effectiveness of curcumin’s bactericidal action due to its ability to diffuse rapidly throughout the cell [32].

For toluidine blue studies, we used dye at a concentration of 100 µM, slightly higher than that reported by Sharma and co-workers (80 µM) [33]. We did not subject the dye to 30 min incubation in the dark, obtaining with the application of the red diode laser an increase of inhibition of up to 100%.

This work, even with the limitations of the in vitro conditions, is a further confirmation of the efficacy of APDT (Antimicrobial Photodynamic Therapy) against bacteria in the attempt to fight bacterial resistance to antibiotics [34,35,36,37] by using products and techniques that are safe and easy.

## 5. Conclusions

The data obtained from this study show that *Streptococcus mutans* is susceptible to different photosensitizers if coupled with the proper diode laser wavelength. In this way, PDT may be very useful for the treatment of infectious diseases, particularly considering the phenomenon of bacterial resistance, which is currently observed with increasing frequency. Moreover, PDT is a simple and safe technique and the therapeutic protocols are feasible in in vivo conditions, without side effects or contraindications and requiring only a few minutes of low-power laser irradiation. These characteristics are and will increasingly become, in our opinion, the key to success in antimicrobial therapy in the future.

## Figures and Tables

**Table 1:** Effect of red laser (650 nm) application on bacterial suspensions with and without toluidine blue.

Samples		Mean CFU	SD	% Inhibition vs. Control
Negative control		1064	13	-
Toluidine blue 10 µM		13	5	98.78
Red diode without toluidine blue	Fluence 10 J/cm^2^	1291	29	−21.33
	Fluence 20 J/cm^2^	1099	43	−3.29
	Fluence 30 J/cm^2^	1085	31	−1.97
Red diode with toluidine blue	Fluence 10 J/cm^2^	1	1	99.91
	Fluence 20 J/cm^2^	0	0	100
	Fluence 30 J/cm^2^	0	0	100

**Table 2:** Effect of blue laser (405 nm) application on bacterial suspensions with and without curcumin.

Samples		Mean CFU	SD	% Inhibition vs. Control
Negative control		408	19	-
Curcumin 100 µM		295	60	27.70
Blue diode without curcumin	Fluence 10 J/cm^2^	347	39	14.95
	Fluence 20 J/cm^2^	242	98	40.69
	Fluence 30 J/cm^2^	261	100	36.03
Blue diode with curcumin	Fluence 10 J/cm^2^	86	49	78.92
	Fluence 20 J/cm^2^	15	3	96.32
	Fluence 30 J/cm^2^	3	2	99.26

**Table 3:** Effect of green laser (532 nm) application on bacterial suspensions with and without erythrosine.

Samples		Mean CFU	SD	% Inhibition vs. Control
Negative control		572	30	-
Erythrosine 100 µM		102	27	82.17
Green diode without erythrosine	Fluence 10 J/cm^2^	385	6	32.69
	Fluence 20 J/cm^2^	342	31	40.21
	Fluence 30 J/cm^2^	355	7	37.94
Green diode with erythrosine	Fluence 10 J/cm^2^	0	0	100
	Fluence 20 J/cm^2^	0	0	100
	Fluence 30 J/cm^2^	0	0	100
